# A short-term association between hospitalizations for mental disorders and ambient temperature in Japan: an ecological study using the LIFE Study data

**DOI:** 10.1265/ehpm.25-00377

**Published:** 2026-02-20

**Authors:** Tasuku Okui, Hiroaki Fukushima, Megumi Maeda, Futoshi Oda, Naoki Nakashima, Haruhisa Fukuda

**Affiliations:** 1 Medical Information Center, Kyushu University Hospital, Fukuoka, Japan; 2 Department of Medical Informatics, Faculty of Medical Sciences, Kyushu University, Fukuoka, Japan; 3 Department of Health Care Administration and Management, Kyushu University Graduate School of Medical Sciences, Fukuoka, Japan; 4 Center for Cohort Studies, Kyushu University Graduate School of Medical Sciences, Fukuoka, Japan

**Keywords:** Japan, Ambient temperature, Mental disorders, Health insurance data, Multivariate meta-analysis, Distributed lag non-linear model

## Abstract

**Background:**

Few studies have investigated the association between ambient temperature and the risk of mental disorders in Japan. In this study, we investigated a short-term association between the risk of hospitalizations for mental disorders and ambient temperature using municipal health insurance data.

**Methods:**

We used the data of the Longevity Improvement & Fair Evidence Study in Japan, and the data of 17 municipalities were employed in the analysis. The daily number of hospitalizations for schizophrenia, depressive disorders, and anxiety disorders was used as the outcome variable. The time-stratified case-crossover design was employed in this ecological time-series study, and a distributed-lag non-linear model using a conditional quasi-Poisson regression model was employed to investigate an association between ambient temperature and hospitalizations for the abovementioned mental disorders. The model was applied to each municipality, and a multivariate meta-analysis was conducted to pool the results of municipalities. In addition, subgroup analyses by sex and age groups were conducted, and temperature-related attributable fractions of the mental disorders were also calculated.

**Results:**

The results of the overall cumulative effect of ambient temperature on hospitalizations for mental disorders indicated that the risk ratio (RR) tended to increase with an increase in temperature regardless of the type of mental disorder. An analysis by sex indicated that the RR tended to increase with an increase in temperature regardless of sex. In addition, an analysis by age group indicated that an increase in RR with increasing temperature was more evident in persons aged <65 years compared to those aged ≥65 years regardless of mental disorders, and that the temperature-related attributable fractions were also higher in persons aged <65 years.

**Conclusions:**

Higher temperatures were associated with a higher risk of hospitalization for mental disorders in Japan, while the degree of the association differed by age group.

**Supplementary information:**

The online version contains supplementary material available at https://doi.org/10.1265/ehpm.25-00377.

## Background

Mental disorders negatively affect the quality of life and work productivity of the patients [1, 2]. In addition, they are associated with a higher risk of chronic diseases and lead to substantially reduced life expectancy [3–5]. A global study indicated that the burden of mental disorders has increased between 1990 and 2020 [6]. In addition, another global study demonstrated that the age-standardized burden of mental disorders has declined between 1990 and 2019, while the number of new cases of mental disorders has increased [7]. In Japan, the estimated number of patients with mental and behavioral disorders showed an increasing trend over the decades, increasing from 1.89 million in 1996 to 4.90 million in 2023 [8]. It is important to identify the risk factors for mental disorders and prevent their incidence and aggravation.

Ambient temperature is a determinant of mental disorders and is associated with mental disorders such as schizophrenia, depressive disorders, mood disorders, and anxiety disorders, as well as with mental and behavioral disorders as a whole [9–12]. In these studies, higher and lower ambient temperatures have been shown to be associated with a higher risk of hospital admissions for mental disorders. In addition, the associations between ambient temperature and the risk of mental disorders have been shown to vary depending on an individual’s characteristics. Furthermore, the patterns of the associations between ambient temperature and the risk of mental disorders by sex and age groups vary across studies [12–18]. In Japan, the ambient temperature has been shown to be associated with suicide [19–21]. However, only a few studies have investigated an association between ambient temperature and the risk of mental disorders. A study using monthly data from a university hospital indicated that the number of patients with mental disorders tended to increase with increasing temperature [22]; however, it is important to investigate this association using larger-scale data.

In this study, we investigated a short-term association between hospitalizations for mental disorders and ambient temperature using the health insurance data of municipalities.

## Methods

### Data

We used the data of the Longevity Improvement & Fair Evidence (LIFE) Study, which collected health insurance data of multiple municipalities in Japan [23]. The LIFE study data cover health insurance data of enrollees of the National Health Insurance and the Latter-Stage Elderly Health Care System and the recipients of public assistants. The data of 17 municipalities from six prefectures in Kanto, Chubu, Kinki, and Kyushu regions were used in the analysis, and the names of the municipalities cannot be disclosed. The periods of the data differ across municipalities, spanning from April 2014 to December 2023. The data of each municipality consists of the enrollees of the health insurance and the recipients of public assistants living in that municipality. We analyzed the daily number of hospitalizations for schizophrenia, depressive disorders, and anxiety disorders. We defined admissions for the mental disorders on the basis of the codes of the International Statistical Classification of Diseases and Related Health Problems 10th Revision (ICD10), as were conducted in previous studies [11, 13, 17]. F20, F32–33, and F40–41, were used as the ICD10 codes for schizophrenia, depressive disorders, and anxiety disorders, respectively [24–26]. Information on sex and five-year age groups was available as characteristics of each hospitalization.

Regarding meteorological information, the data from the Japan Meteorological Agency were employed [27]. Specifically, we used information on daily mean temperature, relative humidity, rainfall, and sunshine duration. However, the meteorological data for each municipality were unavailable in many cases. Therefore, we used the meteorological data that were available for each municipality, and the meteorological data of the capital of the prefecture to which each municipality belongs were used for unavailable meteorological data.

### Statistical analysis

**Fig. 1:**
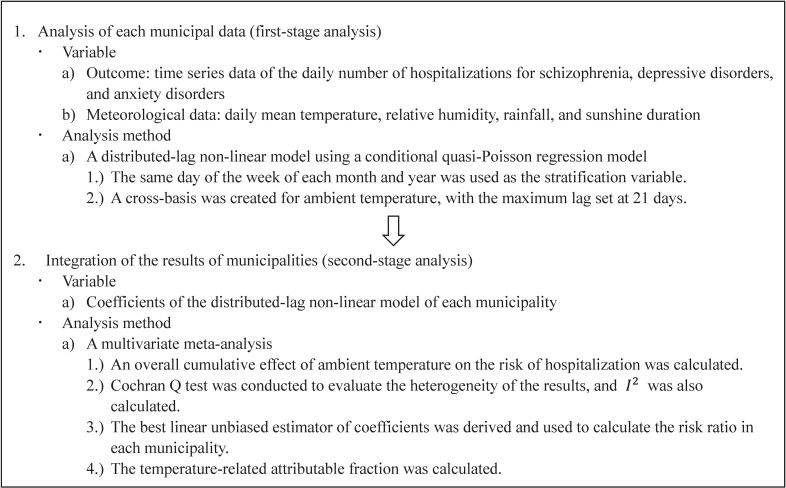
Overview of the analysis in this study

An ecological time-series study was conducted, and Fig. 1 indicates an overview of the analysis in this study. The time-stratified case-crossover design was employed, and a distributed-lag non-linear model using a conditional quasi-Poisson regression model was employed for each municipality to investigate an association between ambient temperature and hospitalizations due to a disease. We counted the daily number of hospitalizations due to the mental disorders in each municipality, and the daily number of hospitalizations was used as the outcome variable. A cross-basis was created for ambient temperature to model the effect of ambient temperature on hospitalization and the delayed effect (lag) [28, 29], and natural cubic spline functions were used for both effects. A one-basis model is often used to investigate a non-linear association between an exposure variable and an outcome variable, while a cross-basis model is used to model a non-linear association between past values (lags) of the exposure variable and an outcome variable additionally, and the cross-basis one models the combined effect of a value of the exposure variable and a lag of the exposure variable on an outcome variable [29]. The maximum lag was set as 7 days in some previous studies investigating an effect of temperature on mental disorders [12, 14, 16], while we confirmed that the sum of the values of the Quasi-Akaike Information Criterion across municipalities decreased by increasing the maximum lag [28, 30]. Therefore, the maximum lag was set at 21 days, which was often used to investigate the delayed effects of cold in some previous studies [13, 31]. Log values of knots were equally spaced in the model, and the number of knots was changed from one to three. The degrees of freedom for the natural cubic spline function of ambient temperature were changed from three to five, and the knots were placed at equal intervals. The number of knots for the lag of ambient temperature and the degrees of freedom for ambient temperature were finally determined based on the sum of the values of the Quasi-Akaike Information Criterion across municipalities [28, 30]. The natural cubic spline functions with five degrees of freedom were also used for relative humidity, rainfall, and sunshine duration, and those were used as explanatory variables in addition to the cross-basis of ambient temperature. The same day of the week of each month and year was used as a stratum in the conditional quasi-Poisson regression model to take into account of the effects of the season and the day of the week [32]. Supplementary Table 1 shows an illustrative table of the time series data used in the analysis using a distributed lag non-linear model. A multicollinearity of the model was checked by the generalized variance inflation factor (GVIF) [33]. Because it was not possible to check the multicollineariy using the conditional quasi-Poisson regression model, it was checked by a quasi-Poisson regression model without the strata. The values of GVIF^(1/(2*Degree of freedom)) were checked [33], and it was confirmed that the degree of multicollinearity was not high.

The model was applied to each municipality, and a multivariate meta-analysis was conducted to pool the results of different municipalities [34]. An overall cumulative effect of ambient temperature (which is the sum of the lag effects) on the risk of hospitalization for each disease was calculated, and the risk ratio (RR) and 95% confidence interval (95% CI) were calculated for each temperature. The minimum-risk temperature between the 1^st^ and 99^th^ percentiles of the overall distribution of ambient temperatures was used as the reference [19, 31]. If the lower bound of the 95% CI exceeded 1, the temperature was defined as exerting a statistically significant effect on the hospitalization compared to the minimum-risk temperature. In addition, the temperature-related attributable fraction (AF) of the hospitalization was calculated [35]. The temperature-related AF indicates the proportion of hospitalizations caused by the temperature, and it quantifies the proportion of hospitalizations that would not have occurred if the temperature had been maintained at the minimum-risk temperature. The best linear unbiased estimator of coefficients derived from the multivariate meta-analysis was used to calculate the RRs in each municipality, and the minimum-risk temperature between the 1^st^ and 99^th^ percentiles of the distribution of ambient temperatures was used as the reference for each municipality for the calculation of RR and AF [19, 31]. The 95% CI of the AF was calculated by Monte Carlo simulations using a multivariate normal distribution, where the best linear unbiased estimators and their variance-covariance matrix were used as mean and variance-covariance, respectively. Moreover, Cochran Q test was conducted in order to evaluate the heterogeneity of the results in the multivariate meta-analysis [36], and *I*^2^, which is an indicator of heterogeneity, was also calculated. A p-value less than 0.05 was judged as statistically significant and the existence of heterogeneity across municipalities.

The analysis was conducted by mental disorder type, and analyses by sex and age group (<65 years and ≥65 years) were also conducted. In the subgroup analysis by sex and age group, the minimum-risk temperature in the total population was used as the reference value when displaying RR in order to align the reference value between subgroups. All the statistical analyses were conducted using R4.5.0 [37] using the car, ggplot2, ggpubr, dlnm, gnm, splines, MuMIn, and mixmeta packages.

## Results

Table 1 indicates the summary statistics of the daily number of hospitalizations and the hospitalization rate for the mental disorders and meteorological data by municipality. The average daily number of hospitalizations and the hospitalization rate differed across the municipalities. The total numbers of hospitalizations for schizophrenia, depressive disorders, and anxiety disorders were 127,537, 120,602, and 56,358, respectively. The proportions of men among hospitalizations for schizophrenia, depressive disorders, and anxiety disorders were 44.4%, 38.6%, and 36.9%, respectively, and those of persons aged <65 years were 21.8%, 21.0%, and 17.7%, respectively.

**Table 1:** Summary statistics of daily number of hospitalizations and hospitalization rate by municipality.

**Municipality number**	**Periods (Year ** **and month)**	**Schizophrenia**		**Depressive disorders**		**Anxiety disorders**	
		
		**Mean daily number (SD)**	Hospitalization rate*	**Mean daily number (SD)**	Hospitalization rate*	**Mean daily number (SD)**	Hospitalization rate*
1	201404–202305	0.9 (1.0)	14.2	1.0 (1.1)	14.6	0.6 (0.8)	8.4
2	201504–202308	0.4 (0.7)	8.5	0.6 (0.8)	12.3	0.3 (0.6)	6.1
3	201503–202308	0.7 (0.9)	11.6	0.7 (0.9)	12.0	0.2 (0.5)	3.5
4	201503–202304	0.7 (0.9)	9.4	0.9 (1.1)	12.4	0.5 (0.7)	6.5
5	201604–202303	17.4 (8.5)	13.2	17.9 (9.2)	13.6	7.9 (4.5)	6.0
6	201503–202311	1.0 (1.1)	11.3	1.1 (1.2)	13.2	0.3 (0.6)	3.8
7	201504–202304	1.4 (1.3)	12.9	1.3 (1.3)	12.6	0.7 (0.9)	6.9
8	201504–202312	4.9 (2.9)	9.3	3.9 (2.5)	7.5	2.6 (1.9)	5.0
9	201704–202304	1.0 (1.1)	15.7	0.9 (1.0)	14.1	0.6 (0.8)	9.2
10	201510–202304	0.4 (0.7)	11.0	0.4 (0.7)	10.1	0.3 (0.6)	8.4
11	201504–202306	1.1 (1.2)	10.7	1.5 (1.5)	14.3	0.8 (0.9)	7.3
12	201801–202304	1.8 (1.6)	10.9	1.6 (1.5)	10.0	0.9 (1.0)	5.2
13	201703–202309	1.3 (1.3)	12.5	1.2 (1.3)	11.8	0.5 (0.8)	4.9
14	201503–202305	6.4 (4.0)	9.3	5.6 (3.7)	8.2	2.8 (2.2)	4.0
15	201704–202207	4.8 (3.0)	12.1	3.1 (2.3)	7.8	0.9 (1.1)	2.3
16	201803–202304	0.3 (0.6)	10.4	0.3 (0.6)	11.1	0.2 (0.4)	6.5
17	201803–202304	5.1 (3.1)	8.0	4.1 (2.7)	6.4	1.2 (1.2)	1.8

SD, standard deviation

*The hospitalization rate indicates the number of hospitalizations per 1,000 person-years. The denominator includes enrollees of the National Health Insurance and the Latter-Stage Elderly Health Care System and the recipients of public assistants, and those who have not visited a hospital in the periods are not included because the data were unavailable. In addition, the person-years for each person were calculated from the difference between the month and year of the first and last appearances in the claims data.

Table 2 indicates the summary statistics of the meteorological data by municipality.

**Table 2:** Summary statistics of meteorological data by municipality.

**Municipality number**	**Periods (Year ** **and month)**	**Mean temperature (°C)**	**Sunshine duration (hours)**	**Relative humidity (%)**	**Rainfall (mm)**

		**Mean (SD)**	**Mean (SD)**	**Mean (SD)**	**Mean (SD)**
1	201404–202305	17.8 (7.7)	5.5 (4.1)	69.5 (12.0)	4.9 (14.9)
2	201504–202308	18.2 (7.8)	5.6 (4.2)	69.8 (11.6)	5.1 (15.4)
3	201503–202308	18.1 (7.8)	5.6 (4.2)	69.7 (11.6)	5.0 (15.4)
4	201503–202304	17.8 (7.7)	5.5 (4.2)	69.5 (11.7)	4.9 (14.9)
5	201604–202303	17.5 (8.1)	6.0 (4.1)	66.0 (11.3)	4.0 (12.3)
6	201503–202311	16.1 (8.2)	5.4 (4.1)	69.7 (11.6)	6.1 (19.4)
7	201504–202304	17.1 (7.7)	5.3 (4.2)	69.6 (11.6)	4.8 (14.9)
8	201504–202312	16.9 (7.5)	5.9 (4.3)	69.6 (16.1)	3.7 (11.5)
9	201704–202304	17.0 (8.2)	5.3 (4.1)	68.7 (11.3)	5.2 (17.2)
10	201510–202304	17.6 (7.8)	5.5 (4.1)	69.2 (11.5)	4.7 (15.1)
11	201504–202306	18.0 (7.7)	5.5 (4.2)	69.7 (11.6)	4.9 (15.0)
12	201801–202304	16.5 (8.6)	5.3 (3.8)	66.6 (11.3)	4.5 (12.9)
13	201703–202309	18.4 (7.9)	5.7 (4.1)	69.0 (11.3)	4.8 (15.5)
14	201503–202305	16.5 (7.7)	5.5 (4.1)	69.3 (16.3)	4.5 (13.3)
15	201704–202207	17.2 (7.5)	5.8 (4.2)	68.7 (15.7)	5.0 (14.6)
16	201803–202304	18.0 (7.6)	5.7 (4.1)	68.6 (11.1)	4.7 (15.6)
17	201803–202304	17.7 (7.2)	6.1 (4.1)	69.6 (14.0)	7.0 (21.7)

SD, standard deviation

**Fig. 2:**
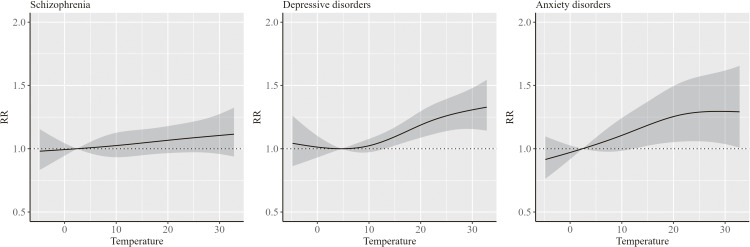
Results of the association between ambient temperature and hospitalization for mental disorders The RR indicates the overall cumulative effect of ambient temperature derived from the multivariate meta-analysis. Solid line indicates the point estimate of the RR, and the shaded areas indicate its 95% confidence intervals. The minimum risk temperatures, which are the reference temperatures of the RRs, for schizophrenia, depressive disorders, and anxiety disorders were 2.4 °C, 4.7 °C, and 2.4 °C, respectively.

Figure 2 shows the results of the association between ambient temperature and hospitalization for mental disorders. The RR tended to increase with increasing temperature regardless of mental disorder type. Statistically significant differences in the risk compared to the minimum-risk temperature were observed for temperatures ranging approximately from 15 °C to 30 °C in depressive and anxiety disorders.

Figures S1–S3 indicate the results of the association between ambient temperature and hospitalizations in each municipality for schizophrenia, depressive disorders, and anxiety disorders, respectively. An increase in the RR with increasing temperatures was observed in most of the municipalities. In contrast, the RR for schizophrenia increased with decreasing temperature in a few municipalities, while the increase in the RR was not statistically significant.

**Fig. 3:**
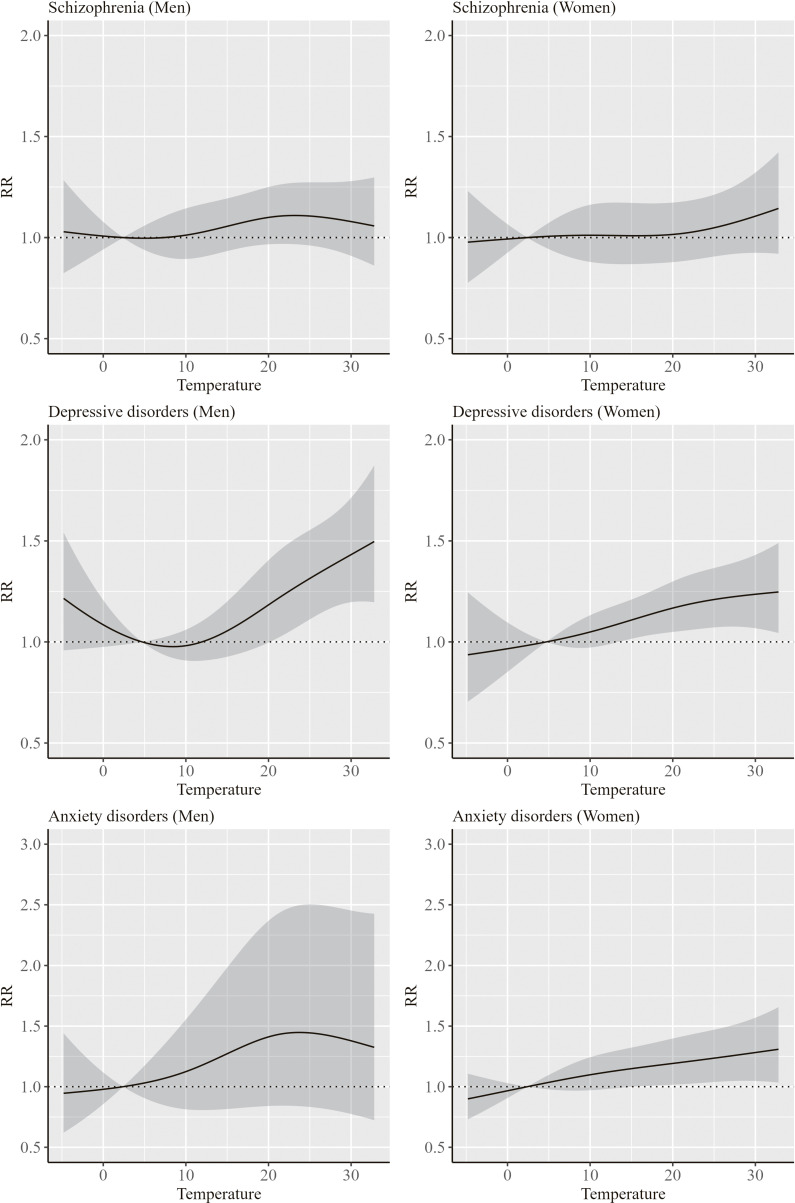
Results of the association between ambient temperature and hospitalization for mental disorders by sex The RR indicates the overall cumulative effect of ambient temperature derived from the multivariate meta-analysis. Solid line indicates the point estimate of the RR, and the shaded areas indicate its 95% confidence intervals. The reference temperatures of the RRs for schizophrenia, depressive disorders, and anxiety disorders were 2.4 °C, 4.7 °C, and 2.4 °C, respectively.

**Fig. 4:**
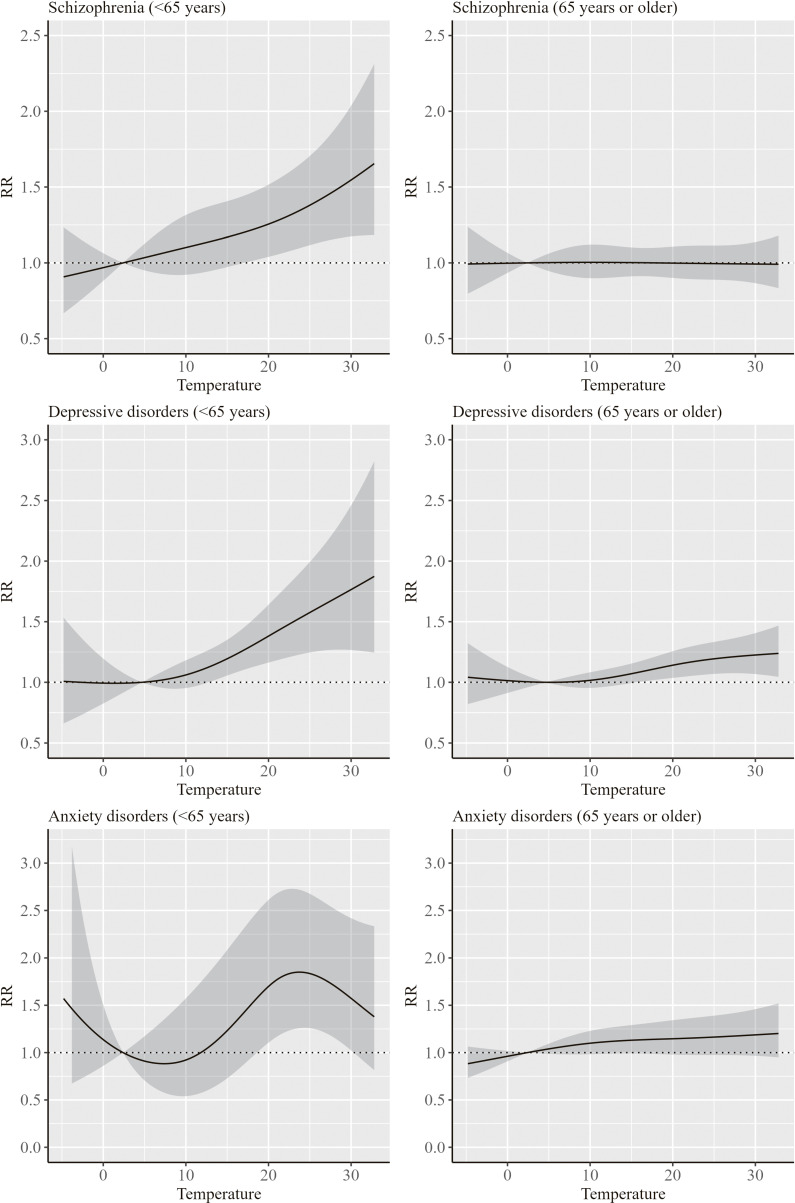
Results of the association between ambient temperature and hospitalization for mental disorders by age group The RR indicates the overall cumulative effect of ambient temperature derived from the multivariate meta-analysis. Solid line indicates the point estimate of the RR, and the shaded areas indicate its 95% confidence intervals. The reference temperatures of the RRs for schizophrenia, depressive disorders, and anxiety disorders were 2.4 °C, 4.7 °C, and 2.4 °C, respectively.

Figures 3 and 4 show the results of the association between ambient temperature and hospitalization for mental disorders by sex and age group, respectively. The RRs for mental disorders showed an increasing trend with an increase in temperature both in men and women. In addition, an increase in RRs for depressive disorders and anxiety disorders was more evident in men at approximately 30 °C and at approximately 20–30 °C, respectively. Furthermore, an increase in RR for mental disorders with increasing temperature was more evident in persons aged <65 years compared to those aged ≥65 years. Particularly, the trend in the RR for schizophrenia largely differed by age groups, with no increase in RR among persons aged ≥65 years.

Table 3 indicates the temperature-related AF by mental disorder type, sex, and age group. We observed a difference in the AF of schizophrenia and anxiety disorders between men and women. Specifically, the AFs of schizophrenia and anxiety disorders in men were 8.60 (95% CI: 2.15, 13.98) and 24.82 (95% CI: 5.21, 33.34), respectively, while those in women were 6.70 (95% CI: −2.49, 13.98) and 16.29 (95% CI: 9.62, 22.30), respectively. In addition, the AFs in persons aged <65 years were larger than for those aged ≥65 years regardless of mental disorder type. Specifically, the AFs of schizophrenia, depressive disorders, and anxiety disorders among persons aged <65 years were 18.13 (95% CI: 7.10, 24.54), 22.40 (95% CI: 12.68, 27.72) and 37.09 (95% CI: −1.90, 49.91), respectively, while those among persons aged ≥65 years were 5.60 (95% CI: 0.01, 9.92), 10.91 (95% CI: 7.27, 13.71), and 14.54 (95% CI: 5.84, 21.00), respectively.

**Table 3:** Results of temperature-related AF by the type of mental disorders, sex, and age group

**Characteristics**	**Schizophrenia**	**Depressive disorders**	**Anxiety disorders**

	**AF (95% CI)**	**AF (95% CI)**	**AF (95% CI)**
Total	6.87 (2.28, 10.05)	12.10 (9.37, 14.40)	15.80 (3.91, 23.78)
Sex			
Men	8.60 (2.15, 13.98)	14.98 (10.85, 18.27)	24.82 (5.21, 33.34)
Women	6.70 (−2.49, 13.98)	14.09 (8.89, 17.75)	16.29 (9.62, 22.30)
Age group			
<65 years	18.13 (7.10, 24.54)	22.40 (12.68, 27.72)	37.09 (−1.90, 49.91)
>=65 years	5.60 (0.01, 9.92)	10.91 (7.27, 13.71)	14.54 (5.84, 21.00)

AF, attributable fraction, CI, confidence interval

Table 4 shows the results of heterogeneity statistics for the multivariate meta-analysis. The p-value of Cochran Q test was statistically significant in persons aged >=65 years with schizophrenia and men and persons aged <65 years with depressive or anxiety disorders, and heterogeneity of the results depending on municipalities existed in those analyses. Notably, *I*^2^ in persons aged <65 years with depressive and anxiety disorders were 35.8% and 66.9%, respectively, and were relatively high.

**Table 4:** Results of heterogeneity statistics for the multivariate meta-analysis.

**Mental disorders**	**Subgroups**	**Cochran Q test**		***I* ^2^**

		**Q statistic**	**p-value**	**%**
Schizophrenia				
	Total	60.6	0.105	20.8
	Men	55.7	0.207	13.8
	Women	65.9	0.044	27.2
	<65 years	57.0	0.175	15.8
	>=65 years	78.7	0.003	39.0
Depressive disorders				
	Total	44.9	0.601	0.0
	Men	67.6	0.033	29.0
	Women	55.8	0.205	14.0
	<65 years	74.8	0.008	35.8
	>=65 years	55.1	0.223	12.9
Anxiety disorders				
	Total	61.2	0.095	21.6
	Men	118.3	<0.001	59.4
	Women	53.4	0.275	10.1
	<65 years	144.9	<0.001	66.9
	>=65 years	63.8	0.063	24.7

## Discussion

Associations between hospitalization for mental disorders and ambient temperature have been demonstrated in this study, and the degree of this association differed by sex and age group.

The RRs of hospitalization for mental disorders tended to increase with increasing temperature. Previous studies have reported an increase in the risk of mental disorders with increasing temperature in other countries [14, 38, 39]. It is pointed out that factors such as medication use and chronic diseases among patients with schizophrenia affect their susceptibility to heat [40]. In addition, some previous studies conducted in China and the United States have shown that the risk of schizophrenia also increased in low temperatures [10, 41]; however, we did not observe such an association. A meta-analysis also demonstrated that lower temperatures increase the risk of schizophrenia [42], with double peaks in the prevalence of schizophrenia in China, which was different from other countries in the Northern hemisphere [42]. Associations between higher temperatures and visits to the mood-related emergency departments and affective disorders have been demonstrated in other countries [9, 43]. In addition, another study using a wearable sensor reported that a high body temperature was associated with the severity of depressive symptoms [44]. Although some studies conducted in China showed that the risk of depressive symptoms or hospital admission for depression increased in lower temperatures [9, 11, 45], an increase in the risk under lower temperatures was not demonstrated in our study. A study conducted in China showed that the risk of anxiety disorders increased at high temperatures [12], and a randomized crossover study also demonstrated a causal relationship between heat and anxiety disorders [46].

We observed that the RR tended to increase with an increase in temperature regardless of sex. Although an increase in RRs for depressive and anxiety disorders was more evident in men at higher temperatures, AFs of depressive disorders due to ambient temperature for men and women were comparable. This is considered to be because there was a difference in RRs for depressive disorders by sex only at high temperatures. In addition, the AF was derived based on the overall cumulative effect obtained from best linear unbiased estimator of each municipality, which is another possible reason. A study conducted in China indicated that male patients with mental disorders were more susceptible to high temperatures [12]. In addition, another study in the United States indicated that an association between extreme heat and visits to the mental health–related emergency department was stronger in men [47]. In the study conducted in China, it is pointed out that women are more sensitive to heat than men, implying that they can take measures against it sooner [12]. In contrast, a study conducted in China demonstrated that women were more vulnerable to higher temperatures in terms of hospitalization for mental disorders [17], and another study conducted in China also showed that women were more vulnerable to lower temperatures in terms of depression [18]. Other studies conducted in China demonstrated that the effect of high temperatures on hospital admissions or hospitalizations for schizophrenia was more pronounced in men [10, 13]. Although AF of schizophrenia was larger in men, there was not large difference in the trends in the RRs for schizophrenia between sex in our study.

The risk of hospitalization for mental disorders also differed by age groups, and the associations between hospitalization for mental disorders and higher temperatures were more evident among persons aged <65 years. Particularly, the trend in the overall cumulative effect of temperature on schizophrenia was almost flat for persons aged ≥65 years. A study conducted in China demonstrated that younger persons were affected more by temperatures in terms of hospitalization for mental and behavioral disorders, attributing fewer outdoor activities among older persons as a possible cause [12]. In contrast, several studies have shown a stronger association between temperature and mental disorders among older persons [13, 14, 48, 49]. In addition, an association between the ambient temperature and the suicide mortality rate was shown to be stronger in persons aged ≥65 years in Japan [19], which did not align with our findings. It is known that there are differences in thermoregulatory responses between young and elderly adults, and elderly persons have more insensitive thermoreceptors [50]. Therefore, it is considered that factors other than the thermoregulatory responses caused the stronger associaiton in the younger age group.

Associations between hospitalization for mental disorders and ambient temperatures have been demonstrated in this study, and the increase in the risk of hospitalization was found to be more prominent among persons aged <65 years. Therefore, alerts or warnings against higher temperatures (approximately 20 degrees Celsius or more) are particularly needed for those persons with mental disorders. In contrast, there was heterogeneity in the results of the associations between ambient temperature and hospitalization across municipalities in persons aged <65 years with depressive or anxiety disorders, and future studies should further explore these regional differences. In addition, it is important to scrutinize the reasons underlying such associations and to investigate the characteristics of persons more vulnerable to higher temperatures. Investigating the association by other individual-level factors such as daily living conditions and use of air conditioners will be meaningful to identify the characteristics of persons more vulnerable to higher temperatures [51].

Our study had limitations. We focused on hospitalizations for mental disorders in this study because the exact dates of the outpatient visits for mental disorders were sometimes uncertain for the outpatient data when the patients visited a hospital multiple times in a month. It is meaningful to investigate the associations using outpatient data in the future in Japan. In addition, because the meteorological data of each municipality was not fully available, we employed the meteorological data of the capital city of the prefecture to which each municipality belongs, for some municipalities in the analysis. Moreover, we used the ICD-10 code to identify patients with mental disorders because a validated technique for identifying patients with those mental disorders from claims data has not yet been established in Japan. Furthermore, it was difficult to distinguish hospitalizations that occurred because of mental disorders from those in which the mental disorders were merely a comorbidity from the data. In addition, the LIFE study data cover health insurance data of enrollees of the National Health Insurance and the Latter-Stage Elderly Health Care System and the recipients of public assistance, while it does not include the other health insurance data, such as the employees’ health insurance association and the Japan Health Insurance Association. Therefore, some selection bias may exist in the study population, particularly among younger people.

## Conclusions

The results of the overall cumulative effect of ambient temperature on hospitalization for mental disorders showed that the RR tended to increase with increasing temperature regardless of mental disorder type. In addition, a subgroup analysis by sex indicated that the RR tended to increase with an increase in temperature regardless of sex. Moreover, a subgroup analysis by age group indicated that an increase in RR with increasing temperature was more evident in persons aged <65 years compared to those aged ≥65 years regardless of mental disorders and that the temperature-related AFs were also higher in persons aged <65 years. Therefore, our study showed that the association between ambient temperature and hospitalization and the difference in the degree of the association by age group.

## Abbreviations

AF: attributable fraction
CI: confidence intervals
dlnm: Distributed lag nonlinear model
GVIF: generalized variance inflation factor
ICD10: International Statistical Classification of Diseases and Related Health Problems 10th Revision
RR: risk ratio

## Supplementary information

**Additional file 1:** **Supplementary table 1.** An illustrative table of the time series data used in the analysis using the distributed lag non-linear model.

**Additional file 2:** **Supplementary fig. 1.** Results of the association between ambient temperature and hospitalizations in each municipality for schizophrenia.

**Additional file 3:** **Supplementary fig. 2.** Results of the association between ambient temperature and hospitalizations in each municipality for depressive disorders.

**Additional file 4:** **Supplementary fig. 3.** Results of the association between ambient temperature and hospitalizations in each municipality for anxiety disorders.

## Declarations

### Availability of data and materials

The data are available from Haruhisa Fukuda upon reasonable requests.
