# Supplementary material for: A short-term association between hospitalizations for mental disorders and ambient temperature in Japan: an ecological study using the LIFE Study data
Source: Environ Health Prev Med. 2026 Feb 20;31:12. doi: 10.1265/ehpm.25-00377 (PMC12950343; doi:10.1265/ehpm.25-00377)
Supplement: Supplementary file 1 — Additional file 1: Supplementary table 1. An illustrative table of the time series data used in the analysis using the distributed lag non-linear model. [file ehpm-31-012-s001.pdf]

Supplementary table 1. An illustrative table of the time series data used in the analysis using the distributed lag non-linear model

Date	Day of the week	Strata	Number of hospitalizations	Temperature (℃)	Sunshine duration (hours)	Relative humidity (%)	Rainfall (mm)
20180301	Thursday	2018-03-Thursday	0	12.4	8.2	45	0
20180302	February	2018-03-February	4	15.6	4.5	20	0
20180303	Saturday	2018-03-Saturday	5	10.3	10.2	80	0
20180304	Sunday	2018-03-Sunday	0	12.4	6	48	93.2
20180305	Monday	2018-03-Monday	5	14.5	0	50	50.2
⋮	⋮	⋮	⋮	⋮	⋮	⋮	⋮
⋮	⋮	⋮	⋮	⋮	⋮	⋮	⋮
20230430	Sunday	2023-04-Sunday	6	12.5	9.3	34	0
